# Immunodynamics: a cancer immunotherapy trials network review of immune monitoring in immuno-oncology clinical trials

**DOI:** 10.1186/s40425-016-0118-0

**Published:** 2016-03-15

**Authors:** Holbrook E. Kohrt, Paul C. Tumeh, Don Benson, Nina Bhardwaj, Joshua Brody, Silvia Formenti, Bernard A. Fox, Jerome Galon, Carl H. June, Michael Kalos, Ilan Kirsch, Thomas Kleen, Guido Kroemer, Lewis Lanier, Ron Levy, H. Kim Lyerly, Holden Maecker, Aurelien Marabelle, Jos Melenhorst, Jeffrey Miller, Ignacio Melero, Kunle Odunsi, Karolina Palucka, George Peoples, Antoni Ribas, Harlan Robins, William Robinson, Tito Serafini, Paul Sondel, Eric Vivier, Jeff Weber, Jedd Wolchok, Laurence Zitvogel, Mary L. Disis, Martin A. Cheever, on behalf of the Cancer Immunotherapy Trials Network (CITN)

**Affiliations:** Division of Oncology, Stanford Cancer Institute, Stanford University Medical Center, 269 Campus Drive, CCSR 1105, Stanford, CA 94305-5151 USA; Division of Dermatology, Department of Medicine, University of California Los Angeles, Los Angeles, CA USA; Division of Hematology/Oncology, Ohio State University, Columbus, OH USA; Medicine, Hematology and Medical Oncology, Mount Sinai Hospital, New York, NY USA; Medicine, Hematology and Medical Oncology, Mount Sinai Hospital, Ruttenberg Treatment Center, New York, NY USA; Department of Radiation Oncology, New York Weill Cornell Medical Center, New York, NY USA; SOM-Molecular Microbiology & Immunology Department, Laboratory of Molecular and Tumor Immunology, OHSU Cancer Institute, Portland, OR USA; INSERM, Integrative Cancer Immunology Team, Cordeliers Research Center, Paris, France; Perelman School of Medicine, University of Pennsylvania, Pathology and Laboratory Medicine, Philadelphia, PA USA; Cancer Immunobiology, Eli Lilly & Company, New York, NY USA; Translational Medicine, Adaptive Biotechnologies Corp, Seattle, WA USA; Immune Monitoring, Epiontis GmbH, Berlin, Germany; Faculty of Medicine, University of Paris Descartes, Paris, France; Department of Microbiology and Immunology, University of California, San Francisco, CA USA; Division of Oncology, Stanford School of Medicine, Stanford, CA USA; Duke University School of Medicine, Durham, NC USA; Human Immune Monitoring Center Shared Resource, Stanford Cancer Institute, Stanford, CA USA; Léon Bérard Cancer Center, Lyon, France; Product Development and Correlative Sciences, Smilow Center for Translational Research, Philadelphia, PA USA; Division of Hematology, Experimental Therapeutics, University of Minnesota, Oncology and Transplantation, Minneapolis, MN USA; Centro de Investigacion Medica Aplicada, Universidad de Navarra, Avda. Pamplona, Spain; Center for Immunotherapy, Roswell Park Cancer Institute, Buffalo, NY USA; Baylor Institute for Immunology Research, Dallas, TX USA; Cancer Vaccine Development Program, Brooke Army Medical Center, Houston, TX USA; Tumor Immunology Program Area, Jonsson Comprehensive Cancer Center, University of California, Los Angeles, CA USA; Adaptive Biotechnologies, Seattle, WA USA; Division of Immunology and Rheumatology, Department of Medicine, Stanford University School of Medicine, Stanford, CA USA; Atreca, Inc, Redwood City, CA USA; Cellular & Molecular Pathology Graduate Program, University of Wisconsin-Madison, Madison, WI USA; Centre d’Immunologie de Marseille-Luminy, Marseille, France; Moffitt Cancer Center, Tampa, FL USA; Immunology Program, Memorial Sloan-Kettering Cancer Center, New York, NY USA; Institut National de la Santé et Recherche Médicale, Institut GrustaveRoussy, Villejuif, France; Tumor Vaccine Group, University of Washington, Seattle, WA USA; Fred Hutchinson Cancer Research Center, 1100 Eastlake Ave N., E3-300, PO Box 19024, Seattle, WA 98109-1023 USA

**Keywords:** Immunotherapy, Biomarker, Clinical trial

## Abstract

The efficacy of PD-1/PD-L1 targeted therapies in addition to anti-CTLA-4 solidifies immunotherapy as a modality to add to the anticancer arsenal. Despite raising the bar of clinical efficacy, immunologically targeted agents raise new challenges to conventional drug development paradigms by highlighting the limited relevance of assessing standard pharmacokinetics (PK) and pharmacodynamics (PD). Specifically, systemic and intratumoral immune effects have not consistently correlated with standard relationships between systemic dose, toxicity, and efficacy for cytotoxic therapies. Hence, PK and PD paradigms remain inadequate to guide the selection of doses and schedules, both starting and recommended Phase 2 for immunotherapies. The promise of harnessing the immune response against cancer must also be considered in light of unique and potentially serious toxicities. Refining immune endpoints to better inform clinical trial design represents a high priority challenge. The Cancer Immunotherapy Trials Network investigators review the immunodynamic effects of specific classes of immunotherapeutic agents to focus immune assessment modalities and sites, both systemic and importantly intratumoral, which are critical to the success of the rapidly growing field of immuno-oncology.

## Background

Immunotherapy has begun to revolutionize cancer treatment, by introducing therapies that target not the tumor, but the host immune system, therapies that possess unique adverse event profiles, and therapies that might cure many types of cancer. The paradigms of drug development, similarly, are in a time of change. Because immune-targeted agents (ITAs) act against tumors by modulating immune cells instead of tumor cells, they do not demonstrate the conventional correlative relationship between toxicity and efficacy. The impact of their unique and diverse mechanisms of action on both drug development and clinical trial design is significant and requires a redefinition of the norms for charting adverse events, antitumor response, and efficacy (Table 1). To encapsulate this shift in paradigm, immunodynamics has been coined as a way to evaluate the impact of a drug or therapy on the immune system.

**Table 1:** Immunoprognostic and Immunotherapeutic Areas

Prognostic	
Immunoscore	
Therapeutic	
Conventional Therapies	
Chemotherapy	
Radiation therapy	
ITAs - Passive	
Cellular Therapy	
Adoptive T and NK cells	
CAR T cells	
ITAs - Active & Specific	
Monoclonal Antibodies	
Tumor-targeting	
Immune-targeting, including checkpoint inhibitors	
Vaccines	
In situ Vaccines	
Cell-based Vaccines	
Dendritic cell–based Vaccines	
Non–cell-based Vaccines	
ITAs - Active & Nonspecific	
Cytokines	
IDO Inhibitors	

*CAR* chimeric antigen receptor, *ITA* immune-targeted agent, *IDO* indoleamine-2,3-dioxygenase

One of the first examples of how immunodynamics plays out in clinical trials emerged in the study of ipilimumab for advanced melanoma. In March 2011, the US Food and Drug Administration (FDA) approved ipilimumab, an antibody against cytotoxic T-lymphocyte-associated protein 4 (anti-CTLA-4), and marked the first, approved, immune checkpoint modulator that significantly improved survival in patients with advanced melanoma. However, ipilimumab also resulted in unique and previously unobserved, immune-related adverse events (irAEs) as well as transient periods of tumor flare or pseudoprogression that preceded clinical response [1]. In addition, nivolumab and pembrolizumab, which block programmed cell death protein 1 (PD-1) and were approved in 2014 in Japan and the United States, demonstrated divergent cycle lengths and raised unanticipated questions about the optimal dosing for immunotherapy. For example, is a single dose level and schedule length optimal for maximal clinical benefit with immunotherapy or do distinct properties, inherent to the checkpoint or the antibody, determine customized schedules and regimens? Before we had an understanding of immunodynamics, in 2009, the clinical development of an agonistic antibody against CD137, 4-1BB, was halted due to severe, potentially immune-related hepatotoxicity. In light of the insights gained from the approvals of ipilimumab, nivolumab, and pembrolizumab, perhaps the 4-1BB trial was halted prematurely.

In addition, identifying a maximum tolerated dose (MTD) might prove less relevant in selecting the recommended Phase 2 dose of an ITA. At this writing, determining the minimum effective dose, the maximum effective dose, and the maximum administered dose seems more relevant. Small molecules achieve tumor reduction by directly targeting cancer cells, and increasing the dose of small molecules is often associated with increasing both the efficacy and toxicity. In this scenario, MTD is often achieved in Phase I trials and helps define what dose should be used for Phase II trials. Immune-targeted agents (ITAs) achieve tumor regression by directly targeting immune cell types not cancer cells. ITAs often do not achieve an MTD since efficacy and toxicity according to dose do not correlate. In these cases, the MAD, which is based on a pre-specified dose range in accordance with Pharmacokinetic data, helps define the Phase II recommended dose. With extensive preclinical models of CTLA-4, PD-1, and 4-1BB therapies, we are faced with these clinical questions and limitations not due to a lack of immunologic hypotheses, but rather due to a lack of adequate assessment of the immune effects in clinical trials. For example, the toxicity profile of ipilimimumab is predictable by its mechanism of action of inhibiting a regulatory component of the immune response, which results in irAEs (e.g., rash, diarrhea, colitis, hypophysitis) from hyperstimulation or overactivation of the immune response in non–tumor tissue. However, the ability of ipilimumab to deplete CTLA-4–expressing regulatory T cells (Tregs) intratumorally and in organs of toxicity is unknown. In addition, PD-1 blockade augments the effector phase of the cluster of differentiation 8 glycoprotein (CD8) T-cell response and increases interferon gamma (IFN-γ) production in patients responding to therapy, but the degree of increased IFN-γ production by PD-1–expressing effector CD8 T cells in PD-L1–positive tumors after 2-week nivolumab vs. 3-week pembrolizumab dosing is unknown. Similarly, 4-1BB agonism directly and indirectly augments CD8 T cells and Th1 response by gene expression in the peripheral blood of patients on urelumab therapy, but the variability by site (i.e., intratumoral vs. intrahepatic) agonism of 4-1BB–positive CD8 T cells after urelumab is unknown.

Therefore, to successfully navigate endpoints in toxicity, efficacy, and dose selection, assessing the mechanisms of action of ITAs in clinical trials is more important than for any prior therapeutic strategy. Despite well-established guidelines on the measurement of pharmacokinetics and pharmacodynamics, no such framework has been established for the effect of therapies on the immune response, or immunodynamics. To improve immunotherapy drug development, the investigators of the Cancer Immunotherapy Trials Network (CITN) have reviewed immunodynamic assays based on the class of ITA being investigated. To organize immune endpoints, we begin with a discussion of immune markers that have prognostic relevance and that should be considered when assessing patient’s tumor-immune characteristics, specifically the *immunoscore*. We then present the immunodynamic endpoints organized by therapies (including conventional therapies) that have immune effects (including chemotherapy and radiation therapy), followed by passive and active immunotherapy agents. Passive immunotherapies include cellular therapies, such as adoptive T, natural killer (NK), and chimeric antigen receptor (CAR) T cells. Active immunotherapies include specific targeted agents, such as monoclonal antibodies—both tumor-targeting and immune-targeting (e.g., checkpoint inhibitors)—and vaccines, including in situ vaccination and non–cell-based and cell-based (e.g., dendritic cell–based) vaccines. Finally, we discuss active immunotherapies that are nonspecific and may augment the immune response in combination or as monotherapies in a generalized fashion, such as cytokines and indoleamine-2,3-dioxygenase (IDO) inhibitors (Table 2).

**Table 2:** Immunodynamic endpoint assessment

Immuno-prognostic Immunotherapeutic Area	Immunodynamic Endpoint	Method of Assessment	Site of Assessment
Prognostic			
Immunoscore	T-cell infiltrate	IHC: 1. CD8 2. CD45RO	Tumor
Therapeutic			
Conventional Therapies			
Chemotherapy	Immunogenic Cell Death	IHC: 1. phosphorylated eIF2α 2. nuclear HMGB1 (late apoptosis-related marker) 3. LC3-B (autophagosome-related marker) 4. Mx1 and TLR3 (IFN signature) 5. CD8/Foxp3 or CD8/CD68 ratios	Tumor
		Gene expression analyses: 1. Cxcl10 2. IFNb 3. TLR3	Tumor
Radiation therapy	Tumor immunogenicity	Gene expression analysis: 1. Immunologic constant region (ICR)	Tumor
	Radiation induced T and NK activation	IHC: 1. MICA	Tumor
		ELISPOT: 1. sMICA 2. anti-sMICA antibodies	Peripheral Blood - Serum
	Memory T cell response	T cell receptor (TCR) repertoire analysis	PBMCs
ITAs - Passive			
Cellular Therapy			
Adoptive T and NK cells	Quantification of adoptive cell population	Flow cytometry–based or PCR-based assessment of unique label in adoptive cell population	PBMCs
Chimeric Antigen Receptor (CAR) T cells	Phenotype of adoptive cell population	Flow cytometry based or PCR based assessment of phenotype of adoptive cell population (see Tables 3 and 4; activation and inhibitory markers including ICOS, 4-1BB, PD-1, PD-L1, OX-40, LAG-3, GITR, VISTA, LIGHT)	PBMCs
	Function of adoptive cell population	Flow cytometry based (perforin, granzyme, intracellular cytokine expression including IFN-γ), ELISPOT, in-vitro cytotoxicity (ie chromium release) or PCR based assessment of phenotype of adoptive cell population after antigen-specific (preferred, ie autologous tumor cells or tumor peptide pulsed T2 cells) or nonspecific stimulation (ie CD3/CD28, PMA, or ionomycin)	PBMCs
	Toxicity	Comprehensive cytokine assessment, CRP	Peripheral Blood - Serum or plasma
ITAs - Active & Specific			
Monoclonal Antibodies			
Tumor-targeting	Target antigen expression in tumor	IHC, multicolor IF, and/or in-situ gene expression of target antigen of mAb on tumor (CD20, HER2, EGFR)	Tumor (primary, metastatic and circulating disease)
	FcR polymorphism	FcR genotype (FcgRIIIA, FcgRIIA)	Peripheral blood – Mononuclear cells (genomic DNA)
	Phenotype and function of immune (T and NK cell) response	T cell receptor (TCR) repertoire analysis Flow cytometry based or PCR based assessment of phenotype of T and NK cells (see Tables 3 and 4) and flow cytometry based (perforin, granzyme, intracellular cytokine expression including IFN-γ), ELISPOT, in-vitro cytotoxicity (ie chromium release) or PCR based assessment of phenotype T and NK cells after antigen-specific (preferred, ie autologous tumor cells or tumor peptide pulsed T2 cells) or nonspecific stimulation (ie CD3/CD28, PMA, or ionomycin)	PBMCs
	Systemic cytokine response	Comprehensive cytokine assessment (IL-6, IFN-γ, IL-10) and serologic assessment (sIL-2Rα, MCP)	Peripheral Blood - Serum or plasma
Immune-targeting, including checkpoint inhibitors	Target expression in tumor microenvironment	IHC, multicolor IF, and/or in-situ gene expression of target on tumor and stroma (CTLA-4, PD-1, PD-L1, OX-40, 4-1BB, LAG-3, GITR, CD40)	Tumor
	Tumor infiltrating immune response	IHC: 1. Quantity and phenotype of tumor-infiltrating lymphocytes (TILs) 2. CD8 effector: CD4 regulatory T cell ratio	Tumor
	Memory T cell response	T cell receptor (TCR) repertoire analysis	PBMCs
	Clinical response	CRP, LDH, WBC, ALC, MDSCs	Peripheral Blood - Serum or plasma
	Toxicity	Comprehensive cytokine assessment	Peripheral Blood - Serum or plasma
Vaccines			
In-situ Vaccines Cell-based Vaccines	Target antigen expression in tumor	IHC, multicolor IF, and/or in-situ gene expression of target vaccination antigen on tumor (gp100, MART, Mucin)	Tumor
Dendritic Cell-based Vaccines Non–cell-based Vaccines	T cell response postvaccination in tumor	IHC, multicolor IF (pre/post assessment of ratio of Treg to Teffectors and CD1a, CD8, CD94; CD207 and HLA-DR), and/or in-situ gene expression of intratumoral T cell population	Tumor
	Quantification of T cell response	T cell receptor (TCR) repertoire analysis Flow cytometry based or PCR based assessment of tumor-specific T cells (dimer, tetramer, dextramer)	PBMCs)
	Phenotype of T cell response	T cell receptor (TCR) repertoire analysis Flow cytometry based or PCR based assessment of phenotype of tumor-specific T cells (see Table 3)	PBMCs)
	Function of T cell response	Flow cytometry based (perforin, granzyme, intracellular cytokine expression including IFN-γ), ELISPOT, in-vitro cytotoxicity (ie chromium release) or PCR based assessment of phenotype of tumor-specific T cells after antigen-specific (preferred, ie autologous tumor cells or tumor peptide pulsed T2 cells) or nonspecific stimulation (ie CD3/CD28, PMA, or ionomycin)	PBMCs
	Humoral response Systemic cytokine response	ELISPOT tumor/antigen antibody response Comprehensive cytokine assessment (GM-CSF)	Peripheral Blood - Serum or plasma
ITargeted Agents Active & Nonspecific			
Cytokines	Intratumoral immune response	IHC, multicolor IF, and/or in-situ gene expression of intratumoral lymphocyte (T, B, and NK cell) population	Tumor
	Phenotype and function of immune(T and NK) response	T cell receptor (TCR) repertoire analysis Flow cytometry based or PCR based assessment of phenotype of T and NK cells (see Tables 3 and 4) and flow cytometry based (perforin, granzyme, intracellular cytokine expression including IFN-γ), ELISPOT, in-vitro cytotoxicity (ie chromium release) or PCR based assessment of phenotype T and NK cells after antigen-specific (preferred, ie autologous tumor cells or tumor peptide pulsed T2 cells) or nonspecific stimulation (ie CD3/CD28, PMA, or ionomycin)	PBMCs
	Systemic serologic and cytokine response	Comprehensive cytokine assessment (IL-6, IFNγ, IL-10) and serologic assessment (sIL-2Rα, MCP)	Peripheral Blood - Serum or plasma
IDO Inhibitors	IDO expression in tumor	IHC and/or in-situ gene expression of IDO1	Tumor
	Inhibition of IDO1 based on Kyn/Trp ratio	Kyn/Trp level and IHC of DC maturation status (CD80, CD86)	Tumor & Peripheral Blood - Serum or plasma
	Phenotype and function of immune (T and NK cell) response	T cell receptor (TCR) repertoire analysis Flow cytometry based or PCR based assessment of phenotype of T and NK cells (see Tables 3 and 4) and flow cytometry based (perforin, granzyme, intracellular cytokine expression including IFN-γ), ELISPOT, in-vitro cytotoxicity (ie chromium release) or PCR based assessment of phenotype T and NK cells after antigen-specific (preferred, ie autologous tumor cells or tumor peptide pulsed T2 cells) or nonspecific stimulation (ie CD3/CD28, PMA, or ionomycin)	PBMCs

*ALC* absolute lymphocyte count, *CRP* C-reactive protein, *DC* dendritic cell, *IDO* indoleamine-2,3-dioxygenase; *IFN-γ* interferon gamma, *LDH* lactate dehydrogenase, *MCP* Monocyte Chemotactic Protein-1; *MDSC* myeloid-derived suppressor cells, *PBMC* peripheral blood mononuclear cells, sIL-2Rα, soluble IL-2 receptor-alpha, *WBC* white blood cell count

## Immunodynamics

### Prognostic, immunoscore

#### Bernard A. Fox

ITAs, by definition, modulate the immune response with systemic and local effects. Assessment of the peripheral blood, may be, but is not always, reflective of the changes within the tumor. Though serial tumor biopsies are therefore of fundamental value in monitoring the effects of novel ITAs, the baseline tumor sample may also portend prognostic significance, as the tumor-immune infiltrate has long been associated with improved outcomes. In 2006, Galon and colleagues, using digital imaging and image quantification software [2], reported a strong and highly significant correlation in colon cancer between increased survival and the presence of immune cell densities (CD3^+^, CD8^+^, Granzyme B^+^, CD45RO^+^ cells) at the invasive margin and center of the tumor. A subsequent study further substantiated these findings in a larger cohort and correlated strong infiltration with disease-free, disease-specific, and overall survival [3]. Importantly, objective assessment of T-cell infiltrates (CD8^+^ and CD45RO^+^ cells), or the immune score was a significantly better prognostic biomarker than tumor-node-metastasis (TNM) staging, recognition of the central role immunity plays in this disease [4]. The observation that immune infiltrates are associated with improved outcomes is not limited to colon cancer. More than 100 publications have reported associations between immune infiltrates and improved outcomes for patients with at least 18 different cancer histologies [5]. Currently the Society for Immunotherapy of Cancer is leading an international effort to validate these findings in a retrospective evaluation performed at 23 centers in 17 countries on tissues from 5000 patients [6].

If applied, determination of the immunoscore is obtained on formalin-fixed paraffin-embedded (FFPE) tissue that has both a portion of invasive margin and tumor center, and the greatest degree of immune infiltrate is selected. Automated immunohistochemistry (IHC) is performed on 2 serial sections with one slide stained with anti-CD45RO antibody and one slide with anti-CD8 antibody [3, 7]. As detailed in the following sections, measurement of the immunodynamic effects within the tumor site extend beyond assessment of the T-cell infiltrate. The additional analysis of the *immunoprofile* of tumors will likely identify other prognostic markers that may be histology dependent. For example, tumor-infiltrating myeloid cells have been associated with poor prognosis in some cancers [8] as well as markers for antigen-presenting cells, B cells, Tregs, and activation and inhibitory or suppressive molecules [9]. It is believed that the immunoprofile correlates with the mutational status of a patient’s tumor: high mutational status would be expected to result in a strong immunoprofile. A limitation of these assessments is the potential heterogeneity of a given tumor, which could alter immune infiltrates of the primary or metastatic sites. Tumor heterogeneity is widely recognized as a hurdle for cancer immunotherapy [10] and may limit the current strategy for immunoprofiling on a single specimen or biopsy due to the confounding nature of intratumor heterogeneity (i.e., variation within a tumor lesion) and intertumor heterogeneity (i.e., variation between metastatic sites). An alternative approach might be to apply novel imaging methods and reagents with short half-lives that identify specific markers and could provide real-time in vivo imaging of an immunoprofile for all metastatic sites simultaneously.

As detailed in the remainder of our review, assessment of the tumor in addition to prognostic value provides predictive import to response to immunotherapy as exemplified by a 3- to 4-fold higher response rate to PD-1/PD-L1 pathway targeted agents among patients with PD-L1–positive tumors. It is anticipated that immunoprofiling of tumors will become a routine evaluation for predictive biomarkers to guide patient selection for specific agents and combination therapies.

## Immuno-oncology treatments

### Therapeutic, conventional therapies

Regulatory approval of ITAs and their development in earlier stages of disease requires comparison to and combination with standards of care. Therefore, determining the immunodynamic effects of chemotherapy, radiation therapy, or immunotherapy/chemotherapy combinations will be limited if the immunodynamic properties of chemotherapy and radiation therapy are not established. Recent studies suggest that both have consequences on the immune response, further supporting the value of evaluating tumor biopsies before and after today’s standard treatment modalities. In the following sections we aim to bridge basic science mechanistic information from murine models and/or human in vitro systems with supportive evidence from patients enrolled in clinical trials.

### Chemotherapy

#### Guido Kroemer and Laurence Zitvogel

The antitumor activity of conventional cancer therapies is dependent, at least in part, on the immune response. However not all therapies induce equivalent immune responses in patients, as the manner of cell death induced may be silent, tolerogenic, or immunogenic [11, 12]. Immunogenic cell death (ICD) inducers including radiation therapy, anthracyclines, and oxaliplatin, as well as unconventional cytotoxic agents (e.g., cardiac glycosides, bortezomib, crizotinib) are endowed with the capacity of stimulating premortem stress responses [13–18]. ICD [19, 20] generates an endoplasmic reticulum (ER) stress response and the activation of the autophagy machinery, both producing a series of damage-associated molecular pattern molecules (DAMPs) culminating in Cxcl10 release promoting the recruitment of intratumoral Th1-Tc1 cells indispensable for tumor control [21, 22].

Monitoring of ICD requires sampling of the tumor itself, ideally, by an excisional biopsy, a core biopsy, or least preferable, a fine-needle aspirate. Immunodynamic monitoring of ICD relies on recent results indicating that the ER stress response, autophagy, and late apoptosis can all be detected in tumor cells at diagnosis and correlate with immune infiltrates and eventually with patient survival. IHC detection of phosphorylated eIF2α (ER stress-response related-marker) [23], nuclear high mobility group box 1 (HMGB1; late apoptosis-related marker) [24], and light chain 3 beta (LC3-B; autophagosome-related marker) [21, 25] are feasible and reliable on FFPE, allowing determining of a relationship between CD8 and forkhead box P3 (Foxp3) or CD8 and CD68 ratios and response to cytotoxic compounds. As our understanding of ICD deepens, the markers that are measured both by IHC and potentially by gene expression that best measure the postchemotherapy immune response will be refined.

### Radiation therapy

#### Silvia Formenti

Combining immunotherapy with radiation therapy, similar to immunogenic chemotherapy, has demonstrated clinical activity [26]. Radiation therapy can induce ICD in a dose-dependent manner and enhance ICD of some chemotherapy agents when used in concurrent regimens [26]. In metastatic cancer, combining immunotherapy with radiation therapy to a metastatic site can convert into systemic responders patients who have previously failed to respond to the same immunotherapy. In such setting, the emergence of an abscopal response (“ab-scopus”, i.e., away from the target, outside the radiation field) appears to be an unequivocal marker of immune response [27]. The main rationale for combining tumor radiation therapy with immunotherapy is to convert the irradiated tumor into an in situ, individualized vaccine [28]. T-cell receptor (TCR) repertoire analysis may provide proof of successful vaccination with the emergence of antigenic spread postradiation, detectable by demonstrating the expansion of memory T cells specific to tumor antigens that were not recognized before radiation therapy [29].

The localized nature of radiation therapy offers a unique opportunity to follow the evolution of the irradiated tumor microenvironment by serial biopsies and to determine the relationship between radiation-induced changes and the development of abscopal effects. For example, biopsies obtained after topical imiquimod treatment of basal cell carcinoma in a randomized, placebo-controlled trial, identified 637 genes induced by imiquimod (a toll-like receptor-7 agonist). Four distinct pathways associated with imiquimod-mediated tumor rejection were identified and led to the definition of the immunologic constant of rejection (ICR) [30]. According to the ICR hypothesis, common effector pathways suggestive of an innate immune infiltrate are upregulated in regressing tumors. A similar signature may develop postradiation and serve as a biomarker to predict which patients will generate antitumor immune responses sufficient to achieve abscopal effects.

A second example of the immunodynamic effects of radiation therapy builds upon the combination with anti-CTLA-4 antibodies [26–28, 31], which requires CD8 T-cell expression of the immune activation marker, NKG2D [32], expressed on NK cells. In patients, blockade of NKG2D is mediated by soluble major histocompatibility complex class I-related chain A (sMICA), which is released by some tumors and reaches high levels in the serum [33]. Therefore, sMICA may be the first biomarker of combination radiation and immunotherapy [34], and as radiation therapy upregulates MICA on the surface of tumor cells, serial biopsies of tumors before and after radiation therapy and ipilimumab are required to assess this immunodynamic endpoint with increased expression of MICA [35].

### Therapeutic*,* immune-targeted agents: passive immunotherapy

#### Cellular therapy, adoptive T cells

##### Carl June, Michael Kalos, and Jan J Melenhorst

Recent technological advances have facilitated the application of synthetic biology to molecularly engineer T lymphocytes by redirecting their specificity to antigens expressed by tumor cells [36]. With this approach, T cells obtained from patients can be manipulated to recognize tumor cells after engineered expression of either CAR or tumor antigen-specific TCR [37]. In both cases, adoptive T cells may bypass immunological tolerance [38], leading to potent and durable antitumor immunity [39–41]. Considerable effort in the field is now focused on trying to identify immunodynamic correlates of bioactivity and efficacy.

 T cell therapy–based measures of immunodynamics are designed to examine and quantify (1) the presence, functionality, and phenotype of infused and persisting T cells (Table 3), and (2) the impact of the infused cells on patient immunobiology and the tumor microenvironment [42]. Importantly, because the therapeutic agents are patient-specific biologic entities, it is essential that biomarker studies for engineered T cell–based approaches also interrogate phenotypic and functional properties of the product, as well as the potential for these cells to expand and differentiate in vivo and manifest potent and long-term antitumor activity. Approaches to study the identity and persistence of T cells include quantitative polymerase chain reaction (PCR) and flow cytometry. Approaches to study the functionality and phenotype of T cells are often based on flow-cytometric methods, with recent advances in the available assays allowing for simultaneous and sensitive evaluation of multiple markers. Approaches to study the effect of T-cell transfer on patient immunobiology, by necessity, are broader and typically involve evaluating the modulation of the milieu of soluble factors (e.g., cytokines, chemokines, growth factors) with immune-regulatory and -effector biologic functions, as well as high-throughput transcriptomic analyses of tumor and T cells obtained from patients.

**Table 3:** Important human T cell receptors in cancer immunotherapy

Receptor family	Cluster of Designation	Ligands
Activating receptors		
T cell receptor complex	CD3	MHC:peptide
Co-receptors		
CD2	CD 2	CD58
CD4	CD4	MHC class II
CD5	CD5	CD72 (?)
CD8	CD8	MHC class I
CD27	CD27	CD70
Activating receptors		
CD28	CD28	B7.1 (CD80), B7.2 (CD86), B7H2 (CD275)
OX40	CD134	OX40L (CD252)
4-1-BB	CD137	CD137L
CD40L	CD154	CD40
DNAM-1	CD226	PVR (CD155), PVR2 (CD112)
TACI	CD267	BAFF (CD257), APRIL (CD256)
BCMA	CD269	BAFF (CD257), APRIL (CD256)
ICOS	CD278	B7H2 (CD275)
GITR	CD357	GITRL (-)
BAFFR	n/a	BAFF (CD257)
Inhibitory receptors		
B7.1	CD80	PDL-1 (CD274), CD28, CTLA4 (CD152)
CTLA4	CD152	B7.1(CD80), B7.2(CD86), B7H2 (CD275)
CD160	CD160	HVEM (-)
CD200R	CD200R	CD200
LAG-3	CD223	MHC class II, other?
2B4	CD244	CD48
LIGHT	CD258	HVEM (-)
BTLA	CD272	HVEM (-)
PDL-1	CD274	PD-1 (CD279)
PD-1	CD279	PD-L1 (CD274), PDL-2 (CD273)
TIM-3	n/a	Galectin-9 (-), other?
TIGIT	n/a	PVR (CD155), PVR2 (CD112)
VISTA (PD-1H)	n/a	B7H4 (-), other?
B7H4	n/a	Unknown
KLRG-1	n/a	E-Cadherin (CD324)

Immunodynamics assays of T-cell therapy to date illustrate a correlation of clinical response with robust in vivo expansion of T cells, intratumoral accumulation [43], and long-term persistence of engineered cells, as well as strong and transient elevation in systemic levels of proinflammatory cytokines, notably systemic interleukin 6 (IL-6) and C-reactive protein (CRP), coincident with the peak kinetics of in vivo T-cell expansion [40, 41], as well as cytokine-release–associated toxicity [44].

### Cellular therapy, adoptive NK cells

#### Don Benson, Lewis Lanier, Jeffrey Miller, and Eric Vivier

NK cells are a population of innate lymphoid cells (ILC) that provide host defense against viruses, bacteria, parasites, and fungus, as well as immune surveillance for cancer. In the peripheral blood of healthy individuals, NK cells comprise between 10–20 % of the lymphocyte population [45]. In humans, NK cells are identified as CD3^-^, CD56^+^ lymphocytes [45]. The NK-cell population found in peripheral blood includes immature NK cells, identified as CD3^-^, CD56^bright^, CD16^-^ lymphocytes, and mature NK cells, which are CD3^-^, CD56^+^, CD16^+^. CD56 and CD16 can also be expressed on subsets of myeloid cells in peripheral blood, which can result in the misidentification of the CD3^-^, CD56^-^, CD16^+^ NK cell. A more definitive identification of this NK-cell subset can be obtained by costaining for CD7 (CD3^-^, CD56^-^, CD7^+^, CD16^+^), which is expressed on all NK cells, but not myeloid cells. NK cells express an extensive repertoire of activating and inhibitory receptors, including KIR2DL, KIR3DL, and CD94-NKG2A receptors, which recognize human leukocyte antigen (HLA) class I molecules as ligands and suppress NK-cell activation. Activating or coactivating receptors on NK cells include CD16, a low-affinity receptor for immunoglobulin G (IgG) that is responsible for antibody-dependent cellular cytotoxicity (ADCC); the NKG2D receptor that recognizes stress-induced ligands MICA, major histocompatibility complex class I-related chain B (MICB), and the UL1-6 binding proteins (ULBP1-6, CD226, DNAX accessory molecule-1; DNAM-1), which recognizes CD112 and CD155; CD244 (2B4), which recognizes CD48, and others. NK cells mediate immune protection by release of perforin, granzymes, cytokines, and chemokines, in particular IFN-γ. Upon activation, NK cells can produce abundant amounts of tumor necrosis factor alpha (TNF-α), granulocyte-macrophage colony-stimulating factor (GM-CSF), IL-10, and chemokine (C-C motif) ligands 3 and 4 (CCL3, CCL4) [46, 47].

Based on the premise that autologous NK cell–based therapies are limited by self-tolerance mediated by inhibitory killer-cell immunoglobulin-like receptor (KIR) recognizing residual self–class I major histocompatibility complex (MHC) molecules on tumors, adoptive transfer of haploidentical NK cells with exogenous IL-2 has been used to treat patients with acute myeloid leukemia (AML), non-Hogdkin lymphoma, and ovarian cancer [48–50]. IL-15 administration may be optimal for stimulating the selective expansion of NK cells and not Tregs. Bispecific killer engagers (BIKEs), which can impart antigen-specific selectivity to NK cells as one created from single-chain Fv specific for CD16 and CD33 (expressed on AML targets) can trigger CD16 on NK cells to kill primary AML and induce cytokine production [51, 52]. The standardization of methods to define and measure NK-cell persistence, expansion, and function after adoptive therapy will facilitate the comparison of different NK-cell products and treatment platforms.

Monitoring the immunodynamics of the NK-cell compartment should include the evaluation of both immunophenotype and function of tumor-infiltrating NK cells (Table 4). Quantifying NK-cell subsets by multiparameter flow cytometry or CyTOF^®^ can be useful to understand the immunophenotype of CD56^bright^ and CD56^dim^ NK cells, and predict their function based on the expression of CD107a (indicating NK-cell degranulation) or intracellular staining for cytokines, usually IFN-γ. Many studies support the notion that NK cells may be integral to the immune response against tumors, justifying particular attention to understand the diversity of this immune subset. The ideal approach to test the functionality of NK cells is to use autologous tumor to determine ex vivo anti-tumor activity.

**Table 4:** Important human NK cell receptors in cancer immunotherapy

Receptor family	Cluster of Designation	Ligand
Activating receptors		
FcγRIIIa (CD16)	CD16	Immunoglobulin G
DNAM-1	CD226	Nectin-2 (CD112), PVR (CD155)
NKG2D	CD314	MICA, MICB, ULBPs
NKp46	CD335	Viral HA, HN
NKp30	CD337	B7-H6
2B4	CD244	CD48
CS1	CD319	CS1
NKG2C	CD158c	HLA-E
KIR2DS1/2/3	CD158h,j	HLA-C2
NKp80	n/a	AICL
Inhibitory receptors		
KIR2DLs	CD158a,b	HLA-C1,C2
KIR3DLs	CD158,e,f,k	HLA-A,B
NKG2A	CD159a	HLA-E
LIR-1	CD85j	HLA-A,B,C
		*(allele-specific HLA-B and HLA-C)*
Adhesion/Trafficking receptors		
N-CAM	CD56	CD56, FGFR1
L-selectin	CD62L	GlyCAM-1, CD34, PSGL-1
PEN-5/PSGL-1	CD162	Selectins
LFA-1	CD11a	ICAMs
LFA-2	CD2	CD58
LFA-3	CD58	CD2
αMβ2	CD11b	fibrinogen, C3bi, ICAM-4

### Therapeutic*,* immune-targeted agents: active immunotherapy – specific

#### Monoclonal antibodies, tumor-targeting

##### Ron Levy

Tumor-directed antibodies are thought to exert their antitumor activity through various mechanisms, including direct killing (signalling-induced apoptosis), complement-dependent cytotoxicity (CDC), and ADCC. To select the optimal immunodynamic monitoring, an awareness of the intrinsic properties of the therapeutic antibody, including its biochemical characteristics (isotype, degree of humanization, mutations, and glycosylation/fucosylation of the Fc fragment) as well as its in vitro mechanisms of action (apoptosis, CDC, ADCC), is required. In patients, studies have demonstrated that the therapeutic efficacy of antitumor antibodies correlated with Fc receptor (FcR) polymorphism in various cancers including lymphoma [53–56], breast cancer [57, 58], and colorectal cancer [59]. Patients harboring FcRs with high affinity for IgG have a better outcome after antibody therapy due to a greater capacity to mediate ADCC. Therefore, FcR polymorphisms should be characterized (at least FcgRIIIa and FcgRIIa) in patients receiving antitumor antibodies. Tumor-directed antibodies can also generate an adaptive immune response, as illustrated by the presence of antitumor T cells in cancer patients after antibody therapy [60–62]. Monitoring the adaptive immune response may be performed using tetramer analysis from the blood and/or directly from the tumor.

### Monoclonal antibodies, immune-targeting (checkpoint inhibitors)

#### Michael Kalos, Ignacio Melero, Antoni Ribas, Paul C Tumeh, and Jedd Wolchok

The success of therapeutics that block the PD-1/PD-L1 inhibitory axis has ushered in a new era in oncology, with these agents likely to become the backbone of cancer therapy in a wide range of cancer types. The immune checkpoints, CTLA-4 and PD-1, are cell-surface receptors that upon binding to their ligands, trigger downstream signaling pathways that serve to inhibit T-cell activity [63, 64]. Therapies that target PD-1 or PD-L1 have shown significant clinical activity in patients with advanced melanoma [65–68], non–small cell lung cancer [69–72], renal cell cancer [73–75], Hodgkin lymphoma [76], head and neck cancers [77], and metastatic bladder cancer [78, 79].

Leading this therapeutic class are anti-PD-1 agents pembrolizumab (Keytruda, Merck & Co), and nivolumab (Opdivo, Bristol-Meyers Squibb Co), which the FDA approved for unresectable or metastatic melanoma in September and December 2014, respectively. These build on a previously approved immune-checkpoint inhibitor, anti-CTLA4 (Yervoy, Bristol-Meyers Squibb Co) in 2011 for the same indication. Based on these findings and the expectation that immunotherapy will impact all fields of oncology, *Science* magazine selected cancer immunotherapy as the breakthrough of 2013 [80].

Critical to understanding how these therapeutic antibodies promote tumor rejection is both the identification of cell types that are altered during blockade and the subsequent mechanistic analysis of how these cell types promote or inhibit tumor regression [81]. Recent investigatory efforts have shown that these antibodies are more clinically effective when preexisting immunity is present in tumors; that is, when tumors have already been identified by the immune system, functionally as a result of local levels of IFN-γ leading to the expression of PD-L1 in the tumor and stroma [75, 82]. Hence, approaches that determine the density, phenotype, and location of immune cell types within the tumor microenvironment and respective PD-L1 expression levels represent one key approach to understanding which cell types and their discrete microenvironments promote or inhibit tumor rejection.

Platforms based on this approach include slide-based, quantitative IHC [5, 82–84] and quantitative multiplexed IHC [85, 86] on tumor samples, as well as assays that reveal in situ gene expression, including transcriptomic profiles on microarrays performed using laser captured microdissected tissue [87, 88]. As the target of interest, PD-L1 may be expressed in the tumor, the stroma, or both, and hence, spatiotemporal information is required, including the invasive tumor margin, stromal components, tumor center, and perivascular niches. As a consequence, optimization in small samples (such as fine-needle aspirates and small core biopsies) represent significant challenges with this approach [65, 66]. PD-1 expression shows superior AUC values and predictive value when compared to single agent PD-L1. Furthermore, the use of different anti-PD1 and anti-PDL1 primary antibodies and the vast number of detection systems available and used by different labs have made it difficult to harmonize IHC read-outs The cellular sources of PD-1 and PD-L1 must be defined and then systemically investigated according to clinical response. Additionally, multiplexed IHC approaches can be used generate to multiparametric, spatially resolved information and capture spatiotemporal interdependencies that are clinically relevant. the presence of constitutive PD-L1 expressing cancer cells without TILs present in the tumor correlates with non-responsiveness to anti-PD1 therapy. The presence of CD68^+^PD-L1^+^ cells at the invasive margin is significantly associated with the presence of interfacing or neighbouring CD8^+^ and PD-1^+^ cells. Determining the relative presence of PD-1 on CD8^+^ and CD8^-^ cells in tumors at baseline remains largely unknown but potentially very important in terms of predicting response. Adding another dimension to understanding the preexisting immunity in the tumor microenvironment is the application of TCR next-generation sequencing based on the unambiguously identifiable TCR-β CDR3 region using genomic DNA from tissue samples that can be used to quantify the diversity and repertoire of the T-cell infiltrate in tumor tissues [89].

In serum, absolute lymphocyte count, baseline eosinophil count, CRP, lactate dehydrogenase, and white blood cell count have been shown to correlate with improved survival in patients receiving ipilimumab (anti-CTLA4) [90–93]. Ongoing studies are investigating the correlative relationships of serum markers with treatment outcome to therapies that block the PD-1/PD-L1 axis with, thus far, the tumor microenvironment expression of PD-L1 demonstrating the tightest relationship with response.

### Vaccines, In Situ Vaccines

#### Josh Brody and Aurelien Marabelle

The in situ vaccination (ISV) strategy consists of intratumoral administration of immunostimulatory products to stimulate antitumor immunity. As with other cancer vaccines, ISV presents tumor-associated antigens in an immunogenic context by using the tumor itself as an antigen source. ISV is actually the first cancer immunotherapy paradigm ever tested, as it has been used in clinical practice since the end of the XIX^th^ century in Europe and in the United States [94]. In the modern era, ISV can be performed with many types of immunostimulatory products, including clinical-grade, live, infectious pathogens such as Bacillus Calmette-Guerin in the treatment of cutaneous metastatic melanoma [95] as well as its peritumoral use in superficial bladder cancer^70^. Toll-like receptor 7 (TLR7) and TLR9 agonists mimicking bacterial nucleic acid have demonstrated their ability to generate antitumoral immunity upon direct administration to vulvar intraepithelial neoplasia [96] and low-grade lymphomas, respectively [97, 98]. ISV using vaccinia viruses genetically modified for preferential infection of cancer cells and expressing GM-CSF have the ability to induce tumor responses and survival benefit in patients with hepatocellular carcinoma [99], and modified herpes virus expressing GM-CSF have generated antitumor immune responses and prolonged disease-free survival in patients with melanoma [100]. Importantly, besides their local, immune-mediated, antitumoral activity, these immunostimulatory products also have the ability to generate a systemic antitumor immune response against distant, noninjected, tumor sites [95, 97, 99].

### Vaccines, cell-based vaccines (dendritic cell–based vaccines)

#### Nina Bhardwaj, Nora Disis, and Karolina Palucka

There are common immunodynamic elements to most agents studied in cancer vaccines, including dendritic cell (DC)–based approaches. DCs can be exploited for vaccination against cancer through various means including (1) nontargeted peptide or protein and nucleic acids–based vaccines captured by DCs in vivo, (2) vaccines composed of antigens directly coupled to anti-DC antibodies, or (3) vaccines composed of ex vivo–generated DCs that are loaded with antigens. Immunodynamic investigation of cancer vaccines has demonstrated that the absolute number of tumor-specific T cells infused or generated with immunization is critical in obtaining a beneficial clinical outcome [101, 102]. Further, recent studies suggest that functional phenotypic changes in immune system cells, such as the induction of polyfunctional T cells, represent a desired endpoint [103]. Flow cytometric–based methodologies are highly quantitative, are reproducible, and can be standardized, and through the use of multiple intracellular and extracellular markers can provide detailed information about both the phenotype and the activation status of the adaptive immune response elicited with immunomodulation [81]. One of the most commonly used quantitative assays in immune-oncology today is enzyme-linked immunospot (ELISPOT) [104]. ELISPOT can enumerate cellular immunity, is possible to standardize, but provides limited functional information [105]. ELISPOT results are limited by lack of reproducibility and requirement for knowledge of antigens recognized or the availability of autologous tumor or tumor lysates with significant clinical material for analysis.

One newer method that may useful is the detailed analysis of the T-cell repertoire via CDR3 spectratyping strategies or deep sequencing with next generation sequencing technology to assess T-cell diversity [106]. The benefit of repertoire analysis is that the method can be quantitative and does not require an a priori knowledge of a specific antigen or depend on T-cell stimulation ex vivo. Repertoire analysis can be accomplished with less than 1 mL of whole blood. While the analysis of the T-cell repertoire is not directly functional, evolution from polyclonality to monoclonality of specific TCRs would suggest an evolving immune response with treatment [101]. Moreover, the development of multiple monoclonal populations could indicate the development of epitope spreading, which has been shown by multiple groups to be predictive of beneficial clinical outcome after cancer vaccination [107]. Unlike flow cytometry or ELISPOT, TCR spectratyping can be applied to direct analysis of tumors as well.

Site of monitoring the immune response postvaccination remains a critical consideration. One of the key locations to examine immune-cancer interactions is the tumor-draining lymph node (TDLN). It has been demonstrated that significant changes in immune-cell populations arise within TDLNs in breast cancer, specifically in CD4^+^ T cells and CD1a^+^ DCs, and such changes strongly correlated with clinical outcome [108, 109]. Data from the site of vaccination supports a direct correlation with antitumor activity and tumor-specific T-cell responses [108]. Delayed-type hypersensitivity (DTH) skin tests have been used to assess cell-mediated immunity in vivo. During a DTH, an antigen (Ag) is introduced intradermally, and induration and erythema at 48 to 72 h postinjection indicate a positive reaction. Lack of a DTH response to a recall Ag is often regarded as an evidence of anergy.

### Vaccines, Non–cell-based vaccines

#### George Peoples and Jeff Weber

Immunodynamic monitoring of peptide-based vaccines provides an advantage to this vaccine strategy. Given that these peptide vaccines stimulate specific T-cell populations with TCRs specific for the peptide-HLA complexes, then clonal expansion or phenotypic assays may be employed. These assays enumerate vaccine-specific T cells by flow cytometry–based testing using peptide-specific dimer, tetramer, or dextramer reagents. Of course, these clonally expanded T cells must also be shown to be functional in cytokine-release or cytotoxicity-measuring assays. Additionally, peptide-based vaccines lend themselves well to DTH monitoring, as these peptides alone are biologically inactive and will only produce a DTH reaction if a peptide-specific cellular immune response has been induced. Immunodynamic studies have demonstrated that vaccine-elicited T cells are heterogeneous with respect to tumor-killing capacity, and only a small subset of vaccine-elicited T cells are efficient at tumor-cell lysis [110, 111]. This is largely due to differences in functional avidity (also known as recognition efficiency): peptide-specific T cells indistinguishable by tetramer staining may differ by up to 1000-fold in peptide requirement for target lysis [111]. Only high-avidity cytotoxic T lymphocytes (CTLs), which may represent 10 % or less of a vaccine-elicited response, could lyse tumor targets [110, 111]. This can be assayed via a flow-cytometric method for rapid assessment of recognition efficiency and functional capacity of antigen-specific T-cell responses [112].

### Therapeutic*,* immune-targeted agents: active immunotherapy – nonspecific

#### Cytokines

##### Kim Lyerly and Paul Sondel

Cytokine-based approaches in cancer immunotherapy have been tested as single agents and combined with other agents including fusion proteins linking cytokines to other therapeutics [113], such as monoclonal antibodies (immunocytokines) [90, 114]. Cytokines may be delivered as proteins or as more innovative strategies such as DNA, enabling in vivo production of the cytokine. IL-2 is the most well-investigated cytokine, though despite extensive investigations and approximately 20 years of post-approval testing, the exact mechanism of its antitumor benefit remains controversial. Thus efforts to analyze in vivo immunodynamics are needed to evaluate known, desired cellular and humoral responses as well as potential antitumor effects [91–95].

Serological parameters after cytokine therapy include downstream cytokines induced by IL-2 that might either be desired or unwanted (ie, IL-6, IFN-γ, IL-10, MCP, etc.), or molecules known to be released by IL-2 in response to activation (such as soluble IL-2 receptor-alpha; Table 5) [115]. More complex functional testing includes the “gold standard” evaluation of the patients’ circulating immune cells for their ability to actually recognize and destroy autologous tumor cells or a cell line derived from autologous tumor [96]. Site of sampling is equally critical, due to the variable systemic effects of cytokine treatment. The parameters measured at the tumor site should include specific tumor and immune changes before and after treatment [97–99, 116]. Noninvasive imaging strategies are just beginning to be incorporated into these monitoring strategies, but could be of future promise [98]. While improved clinical outcomes remain the ultimate goal, immunodynamic assessment will contribute to maximizing the potential of this class of therapeutics, while minimizing the toxicities, and improving the efficiency of finding those strategies that are most effective for patients [117].

**Table 5:** Important human cytokine and chemokine receptors in cancer immunotherapy

Receptor family	Cluster of Designation	Ligands
Cytokine/Chemokine receptors		
IL-1R	CD121a	IL-1β
IL-2Rα	CD25	IL-2 (high affinity)
IL-2/15Rβγ	CD122/CD132	IL-2/15 (intermediate affinity)
IL-7Rα	CD127	IL-7
c-KIT	CD117	stem cell factor
CCR2	CD192	MCP-1 (CCL2)
CCR5	CD195	MIP-1α, RANTES, MCP-3, MIP5
CCR7	CD197	CCL19,CCL21
CXCR1	CD128	IL-8
CXCR3	CD183	CXCL9-11
CXCR4	CD184	CXCL2
CX3CR1	n/a	Fractalkine

### Indoleamine-2,3-dioxygenase inhibitors

#### Kunle Odunsi

The immunoregulatory enzyme IDO catalyzes the rate-limiting step of tryptophan (Trp) degradation along the kynurenine (Kyn) pathway [101]. Both the reduction in local tryptophan concentration and the production of tryptophan metabolites contribute to the immunosuppressive effects of IDO, resulting in multiple negative effects on T lymphocytes notably on proliferation, function, and survival. Tryptophan deprivation also biases the differentiation of naive mouse and human CD4^+^ T cells toward Foxp3-expressing regulatory T-like cells. Moreover, certain tryptophan metabolites activate the aryl hydrocarbon receptor, which has been linked to Treg differentiation [102, 103]. IDO is expressed by activated immune and inflammatory cells in TDLNs and in several human malignancies [118]. IDO1 has been observed to be chronically activated in many cancers, and IDO expression and enzymatic activity correlates strongly with extent of disease and is an independent prognostic factor for reduced overall survival in several malignancies [104, 105].

To assess the impact of IDO inhibition in human clinical trials, a number of correlative immunodynamic endpoints are important to develop [102]. The first is to determine the extent to which the IDO inhibitor alters the Kyn/Trp ratios in the serum and tumor microenvironment of treated patients. The second is to determine immunological endpoints of treatment. Pretreatment and post-treatment biopsies should be analyzed for lymphocyte infiltration by IHC and flow cytometric–based immunophenotyping assays. For IHC, the most critical analyses include IDO1 expression, changes in number, distribution, and phenotype of CD8^+^ and CD4^+^ T cells infiltrating tumor, and changes in patterns of CD4^+^ FoxP3^+^ Treg infiltration. Flow cytometric analyses for the effects of treatment on peripheral blood, tumor lymphocyte numbers, and phenotype (i.e., CD8^+^ and CD4^+^ naïve/effector/central memory subsets, Tregs, and exhaustion markers). Finally, changes in myeloid-derived, suppressor-cell populations can also be assessed by flow cytometry and may be an important endpoint of cytokine treatment [106]. There is currently no widely accepted consensus on how to phenotypically and functionally define this cell population. This represents an area of intense investigation.

## Harmonization and standardization

### Michael Kalos and Jeff Weber

The past few years have seen a profound conceptual shift in how immunodynamic studies are designed and incorporated into clinical trial design, with an increased appreciation for the fundamental contribution of well-executed correlative immune endpoints to the outcome and interpretation of clinical trials.

Informative immunodynamic studies are defined by rationally driven breadth, emphasis on quality, and sample collection schemes based on an appreciation for product-specific biology [119]. With regard to breadth, the major paradigm shift has been an appreciation that, in the context of evaluating agents with pleiotropic and complex biology, studies driven principally or exclusively by the testing of specific hypotheses are unlikely to generate ultimately meaningful and mechanistic data sets. This realization, together with the parallel development of molecular, biochemical, and flow-based platforms that capture large amounts of broad-based immune data, has precipitated a revolution in data generation, the fruits of which are just beginning to become apparent. With regard to quality, the major paradigm shift has been a fundamental acceptance that the establishment of objective quality standards is an essential prerequisite for all but the most preliminary experimentation. Beyond the formal accreditation processes that are in place for clinical laboratories, the issue of quality in correlative studies has been addressed most robustly through consortium or multi-institutional efforts. Such effort has involved either approaches to standardize assays [120, 121] or efforts to harmonize assays across laboratories by defining and ultimately implementing critical platform- and assay-specific variables important for quality [122, 123]. An important parallel effort has involved the development of robust approaches to allow for the collection and analysis of the generated data sets, perhaps best exemplified by the MIBBI (Minimum Information for Biological and Biomedical Investigations), which provides critical conceptual and methodological infrastructure support to this end [124]. With regard to sampling schemes, the major paradigm shifts have involved an appreciation for the temporal kinetics and often transient nature of biomarker responses that necessitate robust and thought-informed sampling, the development of new multiplex assay platforms with minimal sample requirements, as well as the relevance for the need to be able to interrogate relevant and often difficult-to-access biologic specimens such as tumor tissue, lymphatics, and sites of adverse events.

## Conclusion

As the field of immunodynamics continues to mature, application of novel and multidimensional platforms and sensitive assays will enhance the ability to interrogate at a single-cell level and with unprecedented depth to determine the phenotypic and functional attributes of immune cells, providing investigators the possibility of understanding the impact of treatment at the individual-cell level and identify correlates of bioactivity, efficacy, and toxicity. Despite immunotherapy’s current progress toward adoption as a standard of cancer treatment, the majority of cancer is insensitive to or becomes resistant to immune therapy. Only through the adoption of immunodynamic endpoints that are clinically meaningful will immunotherapeutic mechanisms be understood to allow the selection of the most effective front-line agents or combinations, or second-line immune agents if and when immunotherapy fails.

## Abbreviations

ACD: acid citrate dextrose
ADCC: antibody-dependent cellular cytotoxicity
Ag: antigen
AML: acute myeloid leukemia
anti-: antibody to, antibody against
BIKE: Bispecific killer engager
CAR: chimeric antigen receptor (T cells)
CCD: charge-couples device
CCL3: chemokine (C-C motif) ligand 3
CCL4: chemokine (C-C motif) ligand 4
CD8: cluster of differentiation 8 (glycoprotein)
CDC: complement-dependent cytotoxicity
cDNA: complementary DNA
CMI: cell-mediated immunity
CRP: C-reactive protein
CTL: cytotoxic T lymphocyte
CTLA4: cytotoxic T-lymphocyte-associated protein 4
CV: coefficient of variability
CyTOF®: Cytometry by Time of Flight
DAMP: damage-associated molecular pattern molecule
DC: dendritic cell
DIN: the German Institute for Standardization (Deutsches Institut für Normung)
DIN EN ISO/IEC 17025:2005: general requirements for the competence of testing and calibration laboratories
DNAM-1: DNAX accessory molecule-1
DTH: delayed-type hypersensitivity
EDTA: ethylenediaminetetraacetic acid
ELISA: enzyme-linked immunosorbent assay
ELISPOT: enzyme-linked immunospot (assay)
ER: endoplasmic reticulum
FACS: fluorescence-activated cell sorting
FcR: Fc receptor
FDA: (US) Food and Drug Administration
Foxp3: forkhead box P3
GAPDH: glyceraldehyde-3-phosphate dehydrogenase
GLP: Good Laboratory Practice
GM-CSF: granulocyte-macrophage colony-stimulating factor
HLA: human leukocyte antigen
HMGB1: high mobility group box 1
ICD: immunogenic cell death
ICH: International Conference of Harmonisation of Technical Requirements for Registration of Pharmaceuticals for Human Use
ICR: immunologic constant of rejection
IDO: indoleamine-2,3-dioxygenase
IEC: International Electrotechnical Commission
IFN-γ: interferon gamma
IgG1: immunoglobulin G1
IgH: immunoglobulin H
IHC: immunohistochemistry
IL-6: interleukin 6
ILC: innate lymphoid cells
irAE: immune-related adverse event
IRC™: Immune Repertoire Capture™
ISO: International Organization for Standardization
ISV: in situ vaccination
ITA: immune-targeted agent
KIR: killer-cell immunoglobulin-like receptor
Kyn: kynurenine
LC3-B: light chain 3 beta
MHC: major histocompatibility complex
MIBBI: Minimum Information for Biological and Biomedical Investigations
MICA: major histocompatibility complex class I-related chain A
MICB: major histocompatibility complex class I-related chain B
mRNA: messenger RNA
MTD: maximum tolerated dose
NK: natural killer
PBMC: peripheral blood mononuclear cells
PCR: polymerase chain reaction
PD: pharmacodynamics
PD-1: programmed cell death protein 1
PD-L1: ligand 1 of PD-1
PK: pharmacokinetics
qPCR: quantitative polymerase chain reaction
RNAseq: sequencing technology to sequence the repertoire of RNA transcripts in a biologic sample
RUO: research use only
sMICA: soluble major histocompatibility complex class I-related chain A
sMICB: soluble major histocompatibility complex class I-related chain B
TCR: T-cell receptor
TDLN: tumor-draining lymph node
TIL: tumor-infiltrating lymphocyte
TLR7: toll-like receptor 7
TLR9: toll-like receptor 9
TNF-α: tumor necrosis factor alpha
TNM: tumor-node-metastasis (staging)
Treg: regulatory T cell
Trp: tryptophan
TSDR: Treg cell-specific demethylated region
